# Genome‐wide association of Amyloid imaging and plasma biomarkers in Adults with Down Syndrome

**DOI:** 10.1002/alz.092659

**Published:** 2025-01-09

**Authors:** Muhammad Muaaz Aslam, Kang‐Hsien Fan, Lam‐Ha T Dang, Priyanka Gorijala, Ruyu Shi, Eleanor Feingold, Laura Xicota, Ann D Cohen, Benjamin L Handen, Bradley T. Christian, Elizabeth Head, Mark Mapstone, Carlos Cruchaga, Joseph H. Lee, M. Ilyas Kamboh, Alzheimer’s Biomarker Consortium – Down Syndrome (ABC‐DS)

**Affiliations:** ^1^ School of Public Health, University of Pittsburgh, Pittsburgh, PA USA; ^2^ Columbia University Irving Medical Center, New York, NY USA; ^3^ Washington University School of Medicine, Saint Louis, MO USA; ^4^ Gertrude H Sergievsky Center, Columbia University Irving Medical Center, New York, NY USA; ^5^ University of Pittsburgh Alzheimer's Disease Research Center (ADRC), Pittsburgh, PA USA; ^6^ University of Pittsburgh, Pittsburgh, PA USA; ^7^ Department of Medical Physics, University of Wisconsin‐Madison School of Medicine and Public Health, Madison, WI USA; ^8^ University of California, Irvine, Irvine, CA USA; ^9^ Hope Center for Neurological Disorders, Washington University School of Medicine, St. Louis, MO USA; ^10^ Taub Institute for Research on Alzheimer’s Disease and the Aging Brain, Columbia University, New York, NY USA; ^11^ University of Pittsburgh Alzheimer's Disease Research Center, Pittsburgh, PA USA

## Abstract

**Background:**

Adults with Down Syndrome (DS) are at higher risk for Alzheimer's disease (AD) compared to the general population due to trisomy of chromosome 21. Thus, it is important to investigate the role AD‐related biomarkers in adults with DS. Here we have performed genome‐wide association analyses on amyloid‐PET and plasma amyloid levels in the participants of the Alzheimer’s Biomarker Consortium – Down Syndrome (ABC‐DS).

**Method:**

Genome‐wide analyses were performed on 294 non‐Hispanic whites with DS (ranging in age from 25 to 81, Male 54.8%) having biomarker data from amyloid‐PET imaging and plasma levels of Aβ40, Aβ42, and the Aβ42/Aβ40 ratio. The association of two APOE SNPs, rs429358 (E4) and rs7412 (E2) was also examined. We used a joint model of multiple phenotypes to investigate the genetic factors contributing to all amyloid biomarkers levels simultaneously in those participants having the complete phenotypic and genotypic data.

**Result:**

APOE4 was not associated with any Aβ biomarker, but APOE2 was significantly associated with lower Amyloid‐PET levels compared to those without APOE2 (p=0.0002). Genome‐wide association analyses identified multiple genome‐wide significant (GWS) hits including six SNPs for amyloid PET (rs532620170 on chr1, p=2.93E‐09; rs144406757 on chr3, p=3.49E‐08; rs1880432 on chr7, p=4.76E‐08; rs143578940 on chr7, p=4.95E‐09; rs8024654 on chr15, p=1.40E‐08; and rs148455801 on chr19, p=5.98E‐09), one SNP for Aβ40 on chromosome 2 (rs78338676, p=1.50E‐08) and one SNP for Aβ42/Aβ40 ratio also on chromosome 2 (rs75126438, p=1.37E‐08). The multiple amyloid phenotype analysis revealed 14 independent GWS loci on chromosomes 2, 4, 5, 7, 8, 10, 12, 15, and 16, indicating a robust association with both imaging and plasma Aβ biomarkers.

**Conclusion:**

Despite the limited number of sample of DS, we identified a total of 22 potential GWS loci associated with Aβ. These findings highlight the potential of research with the DS population as a valuable resource for identifying genetic elements contributing to AD pathology.

